# Recurrent Fistula between Ileal Pouch and Vagina—Successful Treatment with a Gracilis Muscle Flap

**DOI:** 10.1155/2009/676392

**Published:** 2009-06-14

**Authors:** Feride Aydin, Claus Ferdinand Eisenberger, Andreas Raffel, Alexander Rehders, Stefan Benedikt Hosch, Wolfram Trudo Knoefel

**Affiliations:** Department of General Visceral and Pediatric Surgery, Heinrich-Heine University, 40225 Düsseldorf, Germany

## Abstract

Fistulae between an ileal pouch and the vagina are an uncommon complication of ileal pouch-anal anastomosis following proctocolectomy and mucosectomy in patients with familial adenomatous polyposis coli. Several reports describe the successful use of muscle flaps to close recurrent pouch-vaginal-fistulae (PVF). However, series only contain small numbers and an optimal management has not yet been determined. We report the case of a 26-year old woman with a third recurrence of a PVF after proctocolectomy for treatment of familial adenomatous polyposis in October 2005. Because local approaches failed, definitive closure of the fistula was achieved by interposition of a gracilis muscle flap between the pouch-anal anastomosis and the vagina. The postoperative course was uneventful; the patient was discharged 7 days after surgery and remained free of recurrence and symptomatic complaints for 22 months now. The gracilis muscle flap proved to be an effective method in the treatment of recurrent PVF.

## 1. Introduction

Pouch vaginal fistulae (PVF) after restaurative proctocolectomy with an ileoanal pouch are a serious complication for the patient and a frustrating and diffficult problem for the surgeon. The most common etiologic factor is anastomotic leakage [1], which often leads to septic complications. Early fistulae are most likely associated with technical aspects of surgery, whereas late fistulae result from a more diverse range of causes (e.g., local inflammation, chronic anastomic leakage, and radiation damage) [2, 3].

Symptoms that are attributed to PVF are discharge of flatus and feces through the vagina, recurrent vaginitis, perianal irritation, and incontinence. 

Many procedures have been proposed for PVF such as transvaginal repair, fistulectomy, diversion, and transabdominal procedures such as omentum flap and pre-anal repair. However, these procedures are associated with high recurrence rates ranging from 29–86% in the current literature [2].

According to the high recurrence rate of PVF after conventional surgical intervention, the interposition of muscle flaps was advocated by several groups with encouraging results [2, 4]. The use of muscle flaps for other perianal reconstructive surgery is well established and is mainly applied for the treatment of anal sphincter insufficiency and for perineal reconstruction after previous extended tumor surgery [5, 6]*.*

However this treatment offers new perspectives for patients with PVF as well, in particular after previous attempts at repair have failed. Due to the low incidence of PVF and little experience with this procedure in most of the centers, there is only a small series of patients. Therefore, clear indications for a gracilis muscle flap and operative management are not yet determined. We present our experience with this procedure.

## 2. Case Report

A 26-year-old female otherwise healthy patient with no evidence of Crohn's disease underwent a proctocolectomy with a stapled ileo-anal-pouch anastomosis and protective ileostomy for FAP. The postoperative course was uneventful and there were no signs of an anastomotic leakage. One year later, after closing the protective ileostomy she presented with the first PVF. For fistula-closure a pre-anal-repair and a protective ileal diversion was performed. Intraoperatively the pouch-anal anastomosis was found to be vital, tension-free, and without any abscess cavities or granulation tissue. 

Six months later the fistula recurred. Again a pre-anal-repair was performed with augmentation by placement of an omentum-flap between vagina and ileal pouch. Four months later the third recurrence was approached by a transvaginal fistulectomy. Histopathologically the specimen consisted of fibrotic granulation tissue. Microbiological analysis revealed only bacteria of the gut flora. An antimicrobial treatment was initiated with ceftriaxone and metronidazole for three days.

Eight months later, contrast enema revealed another fistula in the stapled anastomosis area. In order to seal the fistula with a larger amount of healthy tissue and to reconstruct the rectovaginal septum, a different approach with fistulectomy, and interposition of a gracilis muscle flap was chosen.

### 2.1. Gracilis Muscle Flap Interposition Technique for PVF

**Figure 1:**
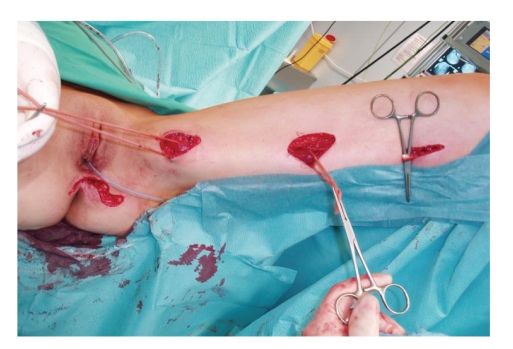
The preparation of the left gracilis muscle flap after perineal incision and closure of the PVF.

**Figure 2:**
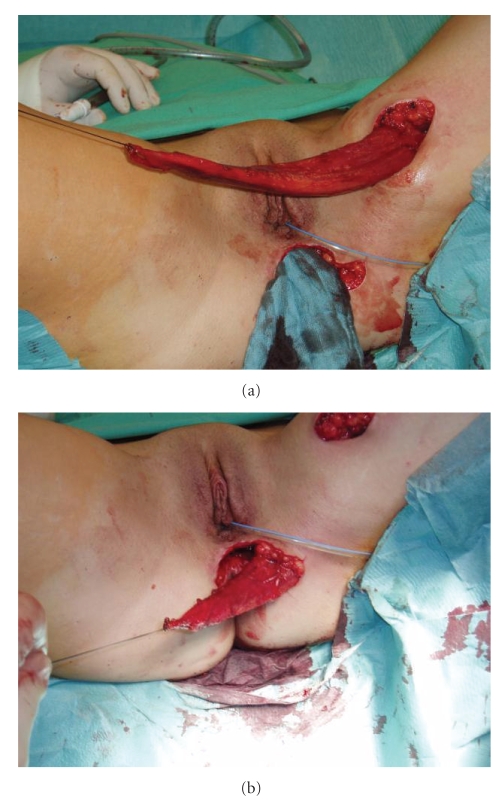
The gracilis muscle flap was identified then elevated trough a subcutaneous tunnel.

**Figure 3:**
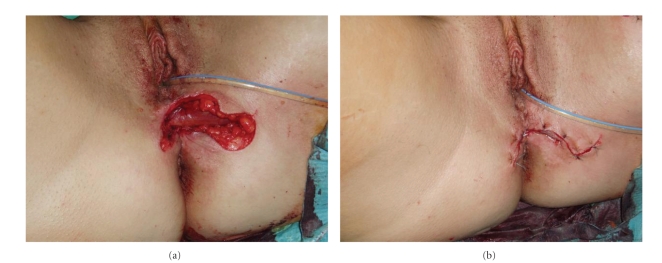
The gracilis muscle flap was interpositioned between the pouch and vagina.

Under general anesthesia the patient was placed in the lithotomy position. After a perineal incision with left lateral extension the PVF was identified. After preparing the fatty tissue the fistula was excised subsequently. The pouch opening and vaginal opening of the fistula was then closed by using absorbable sutures. Now another incision was made at the left medial thigh, the gracilis muscle was identified and secured with a loop (Figure 1). The left gracilis muscle flap was developed after dissecting the tendon from its inserting point at the pes anserinus (Figure 2(a)). Pedicles from the superficial femoral system were ligated and divided. The major pedicle of the medial circumflex artery was localized and carefully preserved. Motor innervation was preserved to prevent a loss in bulk. The muscle flap was then elevated through a subcutaneous tunnel and introduced into the cavity between vagina and pouch (Figure 2(b)), where it was sutured into place and fixed on the anal levator muscle, on the puborectal loop as well as on the pelvic peritoneum (Figure 3(a)). Tension on the vascular pedicle was meticulously avoided. The donor site in the lower extremity was closed in layers over a suction catheter, and the perineal wound was primarily closed (Figure 3(b)).

## 3. Discussion

**Figure 4:**
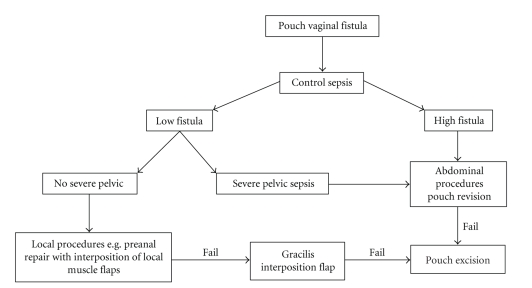
Suggested algorithm for treatment of recurrent PVF: in the case of PVF, the first priority is sepsis control (that is if necessary an ileostomy). Next the type of fistula, high or low should be determined. In the case of a high fistula, an abdominal procedure should be performed. In the case of low fistula the course of therapy depends on the presence or absence of pelvic sepsis. In the case of pelvic sepsis an abdominal procedure should be performed. If there is no severe pelvic sepsis, local procedures should be carried out. These procedures can be repeated. In the case of recurrence, gracilis interposition flap should be performed. Pouch excision should be considered only as the ultimate treatment.

PVF after restorative proctocolectomy represents a rare complication with tremendous impairment of the patient's quality of life; it is also a challenge for the surgeon. These fistulae occur more often in patients with UC (6.3%) than in patients with FAP (1.2%) [7], which might be explained by inflammatory tissue alterations. Another aspect is the type of surgical reconstruction and the way that the pouch-anal anastomosis was performed. Initial reports noted a higher incidence of fistulae after stapled, than after hand-sewn anastomoses [4]. The most common sphincter-saving reconstruction after proctocolectomy and mucosectomy for the treatment of ulcerative colitis (UC) or familial adenomateous polyposis coli (FAP) is the construction of a J-pouch [6]. Generally the J, S, and W-reservoirs are the most common types of pouches that are used. The number of limbs and the amount of small bowel that is used to create them distinguishes the various types of pouches and reconstruction procedures. There are no data available concerning the complication rates and functional outcome of these different types of reservoirs. However, selection of pouch design depends on a variety of factors, including age, patient size, and individual anatomy. Apart from other complications fistulae between an ileal pouch and the vagina occur in 6.3% (range 3.3–16) of female patients with UC and 1.2% with FAP [7]. PVF is generally considered to be a complex fistula, and because of its low incidence, optimal management has not yet been determined; however, many procedures have been proposed [8]. Most of them were adopted from rectovaginal procedures, such as advancement flap or direct repair in layers. Other procedures available are transvaginal repair, fistulectomy and interposition flap. The simplest procedure that has a reasonable chance of success should be tried before more aggressive surgical procedures are considered. The initial approach by transanal or transvaginal repair is frequently recommended [3], but its use as a definitive treatment is in doubt because of its considerably low success rates [9]. Accordingly, the further course of this condition frequently is characterized by recurrent fistulae [7], which is well reflected in the medical history of the presented case. In our case, definitive closure was brought about by a plastic reconstruction with a gracilis flap after an algorithm of several treatments over more than two years had been attempted. The use of muscle flaps to close rectovaginal fistulae is usually performed after the failure of other predominantly local repairs [10, 11]. Most reported series contain small numbers, but the treatments often achieved definitive closure with satisfying long-term results [11]. Therefore the interposition of a gracilis flap can avoid the need of a permanent ileostomy in patients with a course of repeatedly recurrent PVF [12, 13]. Such procedures seem to have been underestimated in the repair of PVF in large series [5]. Nonetheless, interposition of a gracilis flap should be considered, when local treatments have failed to achieve definitive healing (Figure 4). Considering the typically long and distressing course of patients with PVF, the idea to perform a muscle flap interposition as a first-line treatment is tempting, because frustrating recurrences of PVF might be avoided this way and the additional trauma does not lead to a significant increase in morbidity for the patient. However, to support this hypothesis more experience concerning this treatment on a larger cohort of patients is required. At present the algorithm mentioned above is the treatment of choice at our institution (Figure 4).
